# Prevalence and predictors of difficult vascular anatomy in forearm artery access for coronary angiography and PCI

**DOI:** 10.1038/s41598-022-17435-1

**Published:** 2022-07-29

**Authors:** Tobias Roeschl, Anas M. Jano, Franziska Fochler, Mona M. Grewe, Marlis Wacker, Kirstin Meier, Christian Schmidt, Lars Maier, Peter H. Grewe

**Affiliations:** 1 Clinic of Cardiology and Angiology, Klinikum Neumarkt, Neumarkt, Germany; 2 Department of Internal Medicine II, University Hospital Regensburg, Regensburg, Germany; 3 Academic Teaching Hospital, Friedrich-Alexander-University Erlangen, Erlangen, Germany

**Keywords:** Interventional cardiology, Cardiovascular diseases

## Abstract

Transradial access has established as preferred access for cardiac catheterization. Difficult vascular anatomy (DVA) is a noticeable threat to procedural success. We retrospectively analyzed 1397 consecutive cardiac catheterizations to estimate prevalence and identify predictors of DVA. In the subclavian-innominate-aortic-region (SIAR), DVA was causing failure in 2.4% during right-sided vs. 0.7% in left-sided forearm-artery-access (FAA) attempts (χ^2^ = 5.1, p = 0.023). Independent predictors were advanced age [odds ratio (OR) 1.44 per 10-year increase, 95% confidence interval (CI) 1.15 to 1.80, p = 0.001] and right FAA (OR 2.52, 95% CI 1.72 to 3.69, p < 0.001). In the radial-ulnar-brachial region (RUBR), DVA was causing failure in 2.5% during right-sided vs. 1.7% in left-sided FAA (χ^2^ = 0.77, p = 0.38). Independent predictors were age (OR 1.28 per 10-year increase, 95% CI 1.01 to 1.61, p = 0.04), lower height (OR 1.56 per 10-cm decrease, 95% CI 1.13 to 2.15, p = 0.008) and left FAA (OR 2.15, 95% CI 1.45 to 3.18, p < 0.001). Bilateral DVA was causing procedural failure in 0.9% of patients. The prevalence of bilateral DVA was rare. Predictors in SIAR were right FAA and advanced age and in RUBR, left FAA, advanced age and lower height. Gender, arterial hypertension, body mass, STEMI and smoking were not associated with DVA.

## Introduction

Transradial access (TRA) has established itself as the recommended arterial access for coronary angiography and PCI. Compared to transfemoral access (TFA), TRA, or forearm artery access (FAA) in general, is associated with less local bleeding complications and decreased overall mortality^1^. However, difficult vascular anatomy (DVA) can drastically impede successful cardiac catheterization via FAA.

**Figure 1:**
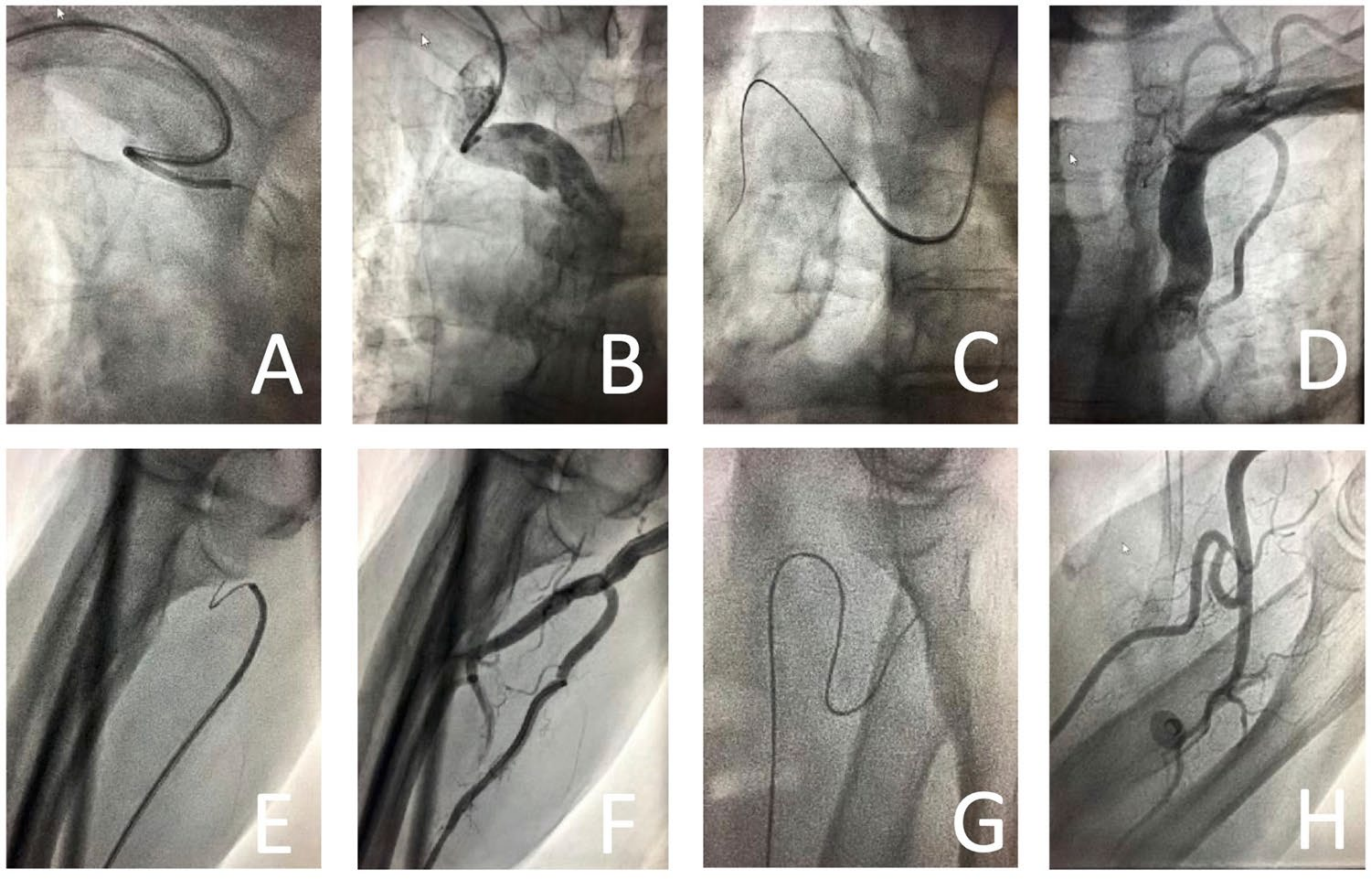
Representative fluoroscopies and angiographies of 2nd degree DVA encountered at our institution within the study period. The figures shown display vascular variants which could not be passed with any of the following guidewires, therefore meeting the definition of 2nd degree DVA: 0.035″ J guidewire (Angiodyn, B. Braun), hydrophilic 0.035″ guidewire (Radifocus Guidewire M, Terumo) and 0.018″ guidewire (Advantage, Terumo). (**A,B**) Severe tortuosity of the innominate artery in a 72-year-old female with suspected CAD. (**C,D**) Aortic arch elongation in a 77-year-old male presenting with NSTEMI. (**E,F**) Right radioulnar loop in a 87-year-old male with suspected CAD. The radioulnar loop became evident only after changing to a RAO 30° projection. (**G,H**) Severe tortuosity of the left radial artery in a 85-year-old female with NSTEMI. *DVA* difficult vascular anatomy, *CAD* coronary artery disease, *NSTEMI* non-ST-elevation myocardial infarction, *RAO* right anterior oblique.

In this study, DVA describes vascular variants impeding guidewire and guide catheter passage in cardiac catheterization. During forearm artery access, vascular variants cluster at two anatomic regions. Firstly, the guidewire and guide catheter must pass the radial-ulnar-brachial region (RUBR), encompassing the proximal radial artery, proximal ulnar artery and distal brachial artery. In this region, radioulnar loops, radial artery stenosis, radial/brachial tortuosity, and atypical arterial branching (e.g., high origin of radial artery) have been identified as potential obstacles previously^2,3^. Secondly, the guidewire must transverse the subclavian-innominate-aortic region (SIAR), encompassing the proximal subclavian artery, the proximal innominate artery, and the transition into the aortic arch, before reaching the ascending aorta and finally the coronary ostia. In this region, subclavian tortuosity, high atherosclerotic burden, subclavian artery occlusion, innominate artery stenosis and aortic arch elongation have been described as anatomical substrates for complex procedures in earlier studies^4,5^. Figure 1 shows representative examples of DVA encountered in our institution during the study period.

Although some technical solutions have been proposed to overcome difficult vascular anatomy^6,7^, DVA is still estimated to be the reason for procedural failure in 2–10.8% of cases^8–10^, and one of the main reasons for crossover to the riskier TFA as has been proven^11^. Besides technical challenges^12^, DVA is also associated with a higher occurrence of vascular complications at the arm, e.g., arterial perforation, arterial dissection, hematoma formation or compartment syndrome^13^.

While the prevalence of vascular variants in right SIAR has been the subject of numerous studies^6,10,14^, data on vascular obstacles in RUBR or at the left arm in general is sparse. This contrasts with an increasing openness for alternative access sites (e.g., left distal radial artery, ulnar artery, brachial artery) to avoid TFA^15,16^.

The main objective of our study was to investigate the prevalence of 1st degree and 2nd degree DVA in RUBR and SIAR for the left and right arm respectively (see Table 1). Another aim was to estimate the prevalence of bilateral DVA necessitating crossover to TFA in a real-life clinical setting. Beyond that, we set out to identify clinical risk factors for difficult vascular anatomy, to provide patient-tailored preoperative planning in future endovascular interventions. Lastly, we aimed to further substantiate our findings by assessing the prevalence of DVA with respect to right vs. left FAA in intraindividual comparisons.

**Table 1:** DVA Classification.

No DVA	Angiographically normal vessel without difficulties to advance a standard 0.035″ J guidewire (Angiodyn, B. Braun) facilitated with leftward rotation of the head and deep inspiration when necessary
DVA	1st degree DVA: Tortuous, stenosed or calcified vessel which could only be passed with a hydrophilic 0.035″ guidewire (*Radifocus Guidewire M,* Terumo) or a 0.018″ guidewire (Advantage, Terumo)
	2nd degree DVA: Severely tortuous, calcified, or occluded vessel which could not be overcome with a guidewire or guide catheter handling was severely impaired resulting in procedural failure and crossover to another access site

## Methods

### Patient selection

In this retrospective, single-center analysis, 1397 consecutive coronary angiographies (CA) with or without percutaneous coronary intervention (PCI), corresponding to 1108 individual patients, between 11/2019 and 12/2020 were analyzed. Cases who required simultaneous right and left heart catheterization were excluded only if this coincided with primary transfemoral access (n = 8). All procedures, where FAA was not the primary access site were excluded (n = 33). In 1328 procedures successful sheath insertion allowed for adequate assessment of DVA in RUBR. In 1306 procedures the guidewire reached the innominate or left subclavian artery allowing for adequate assessment of DVA in SIAR. In 125 individual patients, DVA in RUBR and in 107 individual patients, DVA in SIAR could be assessed bilaterally allowing for intraindividual comparisons between right and left FAA.

### Technical considerations

In our standardized approach, operating physicians were urged to attempt forearm artery access (FAA) for cardiac catheterization whenever possible. Ultrasound-guided arterial puncture followed by standardized forearm angiography including the ipsilateral distal brachial artery was mandatory after sheath insertion (Glidesheath Slender Terumo, 6-French or 7-French, Administration of weight-based dose of unfractionated heparin (50 IU/kg). Guidewires were advanced under continuous fluoroscopic guidance and retrograde angiograms were obtained when necessary. The choice of forearm artery and side used in an individual patient was at the discretion of the operator. By institutional policy, left FAA was mandatory in patients with left internal mammary bypass graft. Arterial access sites included the distal radial artery in the anatomical snuffbox, the proximal radial artery, the ulnar artery, and the anterior interosseous artery. Primary TFA was only performed if inaccessibility of all major forearm arteries was evident from patient history (e.g., dialysis shunts on both forearms) or previous angiographies. In case of failure to complete cardiac catheterization via the primarily chosen forearm artery, operating physicians were principally required to crossover to another artery on the ipsilateral or contralateral forearm by institutional policy (“forearm-only-strategy”). During the study period, cardiac catheterization was performed by six interventional cardiologists with a record of at least 300 ultrasound-guided forearm artery cannulations each. After each case of cardiac catheterization, operating physicians documented the presence and the severity of DVA.

### Difficult vascular anatomy

After FAA for cardiac catheterization was established, vascular anatomy of arm arteries was assessed functionally regarding successful guidewire/-catheter passage. A total of three categories were defined (see Table 1). The prevalence of 1st and 2nd degree DVA was assessed in four regions of interest: RUBR and SIAR of the left and right arm respectively. Only cases, where at least the guidewire reached the region of interest, were included to determine the prevalence of DVA, e.g., if coronary access failed due to a vascular obstacle in right RUBR, this was counted as 2nd degree DVA in right RUBR but not included to determine the prevalence of DVA in right SIAR.

To meet the assumption of independence, as far as possible in a clinical setting, only the first access attempt was included to determine the prevalence of DVA, i.e., if right TRA failed due to e.g., a radial loop and crossover to left TRA was necessary, observations from the left arm were not included in the statistics.

Patients, who had left and right FAA either in the same or a subsequent procedure during the study period, were included for intraindividual comparison analysis to further investigate the association between left- vs. right-sided FAA and DVA.

### Statistical analysis

For statistical analysis, continuous variables were summarized as mean ± standard deviation (SD) and median ± interquartile ranges ([IQR]) for non-normally distributed variables. QQ-plots and the Shapiro–Wilk test were used to assess normality. Rates of interest were reported with 95% confidence intervals respectively. We analyzed the prevalence of 1st -and 2nd degree DVA for each anatomical location respectively. Pearson’s chi-square test was used for comparison of dichotomous data.

Predictors of DVA, encompassing the presence of either 1st or 2nd degree DVA, were evaluated separately for RUBR and SIAR. Clinical predictors were identified as follows: variables that were significant in univariate analysis were included in subsequent multivariable analysis to find independent predictors of DVA. A generalized estimating equations (GEE) binomial model with a logit link and an exchangeable correlation structure was applied to account for correlated observations since some patients had more than one procedure within the study period.

For comparison of paired proportions, McNemar’s exact test was used and odds ratios with central 95% confidence intervals were reported. For each patient, the chronologically first evaluation of DVA was included for each arm respectively. Time spans between intraindividual evaluations were reported as mean ± standard deviation. A two-sided p-value < 0.05 was considered significant.

Due to technical failure, data for estimated glomerular filtration rate was missing in two procedures. Missing data was classified as missing at random and listwise deletion was applied where necessary. All statistical analyses were carried out with Python 3.9 and R 4.0.3. Ethics committee approval was waived for this retrospective analysis (Ethics committee Friedrich-Alexander-University Erlangen, Erlangen, Germany, Number: 20-548_1-Br). All experiments were performed in accordance with the Declaration of Helsinki. Written informed consent was obtained from all participants as part of the admission contract, which is signed by any patient of our hospital. In this contract, the patients agree, that an anonymized retrospective analysis of their data is allowed.

## Results

A total of 1397 procedures of coronary angiography and percutaneous coronary intervention were performed in our institution between mid-November 2019 and December 2020. Eight procedures were excluded as describe above. For subsequent analysis, 33 procedures, where primary TFA or primary transbrachial access was performed, were excluded. The remaining 1356 procedures (97.6%), in which primary FAA was attempted, were further analyzed (see Supplementary Fig. 1). Left FAA was chosen in 745 (54.9%) of cases and right FAA was chosen in 611 (45.1%) of cases. The relative proportions of FAA sites are summarized in Supplementary Table 2. The 1356 procedures consisted of diagnostic coronary angiography in 817 (60.3%) cases and PCI in 539 (39.7%) cases. Clinical indications were suspected coronary artery disease or chronic coronary syndrome in 1121 (82.7%) cases, acute coronary syndrome in 230 (17.0%) cases and “other” in 5 (0.4%) cases. A total of 91 (6.7%) patients presented with ST-elevation myocardial infarction. Median patient age was 71.3 [61.2, 79.4] years with a range from 25 to 95 years. 534 (39.4%) patients were above 75 years of age. Of all patients, 28.3% were female, median weight was 83.0 [73.0, 94.0] kg, mean height was 171.8 ± 9.3 cm, and mean body mass index was 28.4 ± 5.1 kg/m^2^. 86.9% of patients had arterial hypertension, 28.1% had diabetes mellitus, and 25.1% of patients were active smokers. Mean estimated glomerular filtration rate was 74.7 ± 27.3 ml/min/1.73 m^2^ (see Supplementary Table 1).

Primary FAA was attempted in 97.6% of procedures. Crossover to TFA after one or multiple FAA attempts was observed in 2.6% of cases. In 1356 consecutive procedures, where primary FAA was attempted, severe DVA, either in RUBR or SIAR, caused primary FAA failure in 3.3% [95% CI 2.7 to 4.3%] of cases. There were four individual patients, where DVA caused FAA failure on both arms during a single procedure and six individual patients where primary TFA or primary transbrachial access was chosen due to known bilateral 2nd degree DVA from previous angiographies. In 1057 individual patients, the presence of bilateral 2nd degree DVA could be adequately ruled out. Therefore, bilateral 2nd degree DVA was found in a total of 10 out of 1067 individual patients in our study population, resulting in a prevalence of 0.9% [95% CI 0.4 to 1.5%]. Out of 26 procedures, where severe DVA caused FAA failure at one arm, DVA also caused failure at the other arm in 4 cases (15.4% [95% CI 1.5 to 29.3%]).

Out of 125 individual patients, where DVA in RUBR could be assessed bilaterally during the study period, two patients had bilateral 2nd degree DVA in RUBR. Out of 107 individual patients, where DVA in SIAR could be assessed bilaterally during the study period, no patient had 2nd degree DVA in SIAR.

In RUBR, 2nd degree DVA was observed in 2.5% of procedures with right FAA and 1.7% of procedures with left FAA (χ^2^ = 0.77, p = 0.38, power = 0.19). 1st degree DVA was more prevalent in left RUBR compared to right RUBR (11.6 vs. 4.1%, χ^2^ = 23.2, p < 0.001). 2nd degree DVA in SIAR was observed in 2.4% of procedures with right FAA and in 0.7% with left FAA (χ^2^ = 5.1, p = 0.023). 1st degree DVA was more frequently observed in right SIAR compared to left SIAR (13.7 vs. 7.1%, χ^2^ = 14.5, p < 0.001). 

Table 2 summarizes the prevalence of DVA during primary FAA in the procedures under review.

**Table 2:** Prevalence of difficult vascular anatomy (DVA) at the primary FAA site.

	Right arm	Left arm	p-value
**SIAR (n = 1306)**			
No DVA	497 (84.0%)	658 (92.2%)	< 0.001
1st degree DVA	81 (13.7%)	51 (7.1%)	< 0.001
2nd degree DVA	14 (2.4%)	5 (0.7%)	0.023
**RUBR (n = 1328)**			
No DVA	563 (93.4%)	629 (86.8%)	< 0.001
1st degree DVA	25 (4.1%)	84 (11.6%)	< 0.001
2nd degree DVA	15 (2.5%)	12 (1.7%)	0.38

In 1328 procedures successful sheath insertion allowed for adequate assessment of DVA in the radial-ulnar-brachial region (RUBR). In 1306 procedures, DVA could be adequately assessed in the subclavian-innominate-aortic region (SIAR).

During a total of 40 transulnar access (TUA) attempts, including secondary and tertiary access attempts, 2nd degree DVA with respect to the ulnar artery was never reported and was also not observed when 2nd DVA prevented ipsilateral TRA (n = 3) in the first place.

The prevalence of 2nd degree DVA was numerically higher in RUBR compared to SIAR without reaching statistical significance (2.0 vs. 1.5%, χ^2^ = 0.97, p = 0.33, power = 0.34).

**Figure 2:**
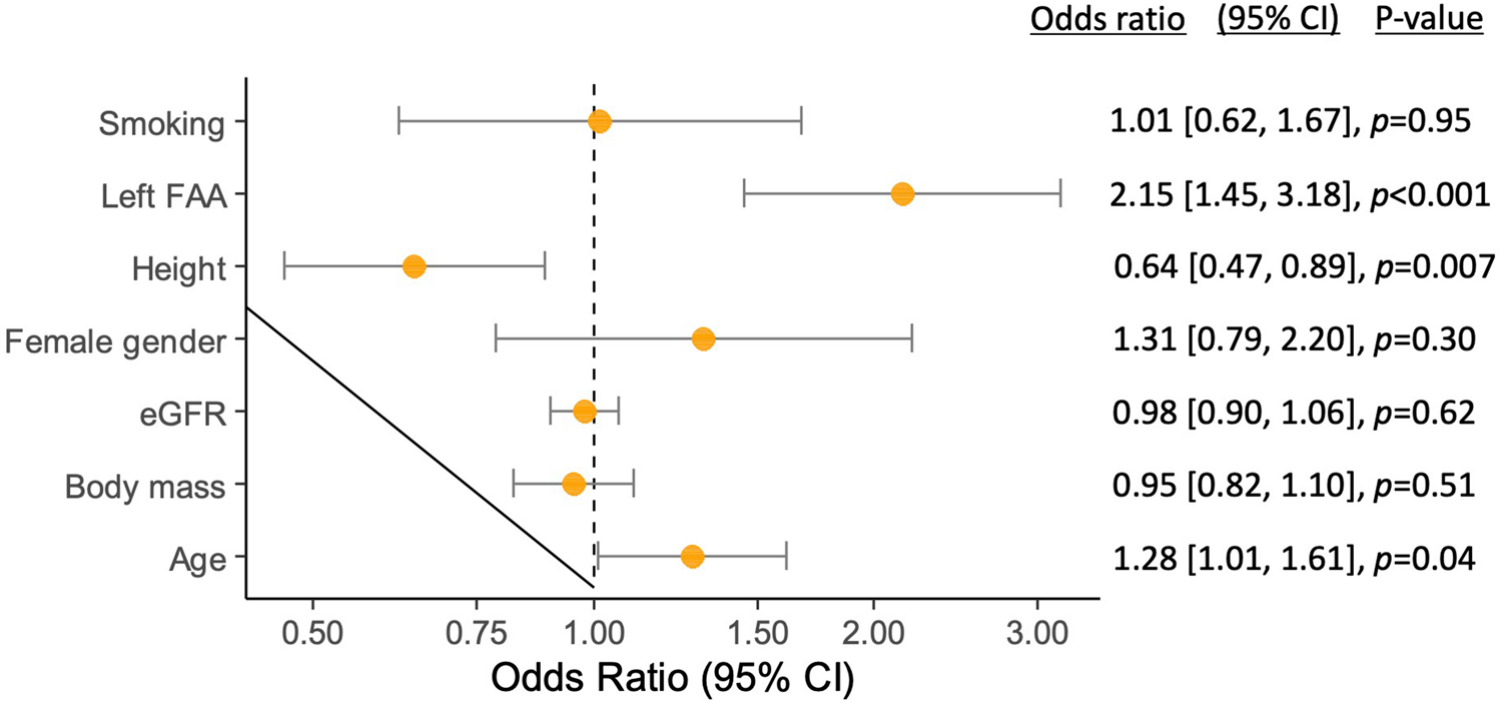
Multivariable logistic regression analysis of DVA in RUBR. Adjusted odds ratios with confidence intervals for difficult vascular anatomy (DVA) in RUBR were illustrated on a logarithmic scale for the following covariates: age (per 10-year increase), body mass (per 10-kg increase), estimated glomerular filtration rate (eGFR; per 10-ml/min/1.73 m^2^ increase), female gender, height (per 10-cm increase), left forearm artery access (FAA) and smoking. *RUBR* radial-ulnar-brachial region.

DVA in RUBR was observed in 136 out of 1328 procedures (10.2%). In procedures, where 1st or 2nd degree DVA in RUBR was observed, patients were significantly older (74.6 [72.7, 77.0] vs. 69.1 [68.5, 70.0] years, p < 0.001), of lower height (166.6 [165.1, 168.0] vs. 172.3 [171.8, 173.0] cm, p < 0.001), of lower body mass (77.4 [74.5, 80.0] vs. 84.8 [83.8, 86.0] kg, p < 0.001), had lower estimated glomerular filtration rates (67.2 [62.8, 72.0] vs. 75.5 [74.0, 77.0] ml/min/1.73 m^2^, p < 0.001), were more frequently female (48.2 [39.8, 56.5] vs. 25.9 [23.4, 28.3]%, p < 0.001), were less frequently current smokers (16.9 [10.6, 23.2] vs. 25.9 [23.4, 28.4]%, p = 0.019) and had more often left FAA (70.1 [62.4, 77.7] vs. 52.8 [50.0, 55.6]%, p < 0.001). The prevalence of DVA in RUBR was not significantly different in patients who presented with STEMI (see Supplementary Table 3). In multivariable analysis, advanced age (odds ratio (OR) 1.28 per 10-year increase, 95% confidence interval (CI) 1.01 to 1.61, p = 0.040), lower height (OR 1.56 per 10-cm decrease, 95% CI 1.13 to 2.15, p = 0.007) and left FAA (OR 2.15, 95% CI 1.45 to 3.18, p < 0.001) remained independent predictors of DVA in RUBR (see Fig. 2).

**Figure 3:**
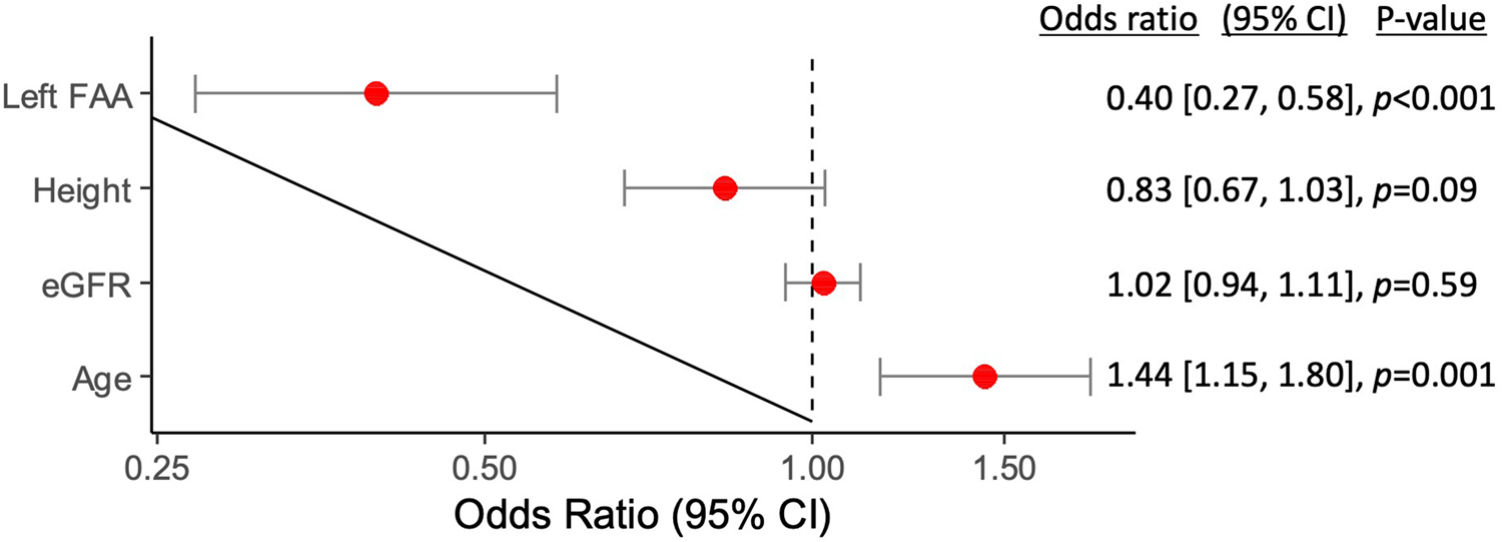
Multivariable logistic regression analysis of DVA in SIAR. Adjusted odds ratios with confidence intervals for difficult vascular anatomy (DVA) in SIAR were illustrated on a logarithmic scale for the following covariates: age (per 10-year increase), estimated glomerular filtration rate (eGFR; per 10-ml/min/1.73 m^2^ increase), height (per 10-cm increase) and left forearm artery access (FAA). *SIAR* subclavian-innominate-aortic region.

DVA in SIAR was observed in 151 out of 1306 procedures (11.6%). In procedures, where DVA in SIAR was observed, patients were significantly older (73.6 [71.7, 76.0] vs. 69.1 [68.4, 70.0] years, p < 0.001), of lower height (169.5 [168.1, 171.0] vs. 172.1 [171.6, 173.0] cm, p = 0.001), had lower estimated glomerular filtration rates (70.6 [66.1, 75.0] vs. 75.2 [73.7, 77.0] ml/min/1.73 m^2^, p = 0.047) and had significantly less often left FAA (37.1 [29.4, 44.8] vs. 57 [54.1, 59.8]%, p < 0.001). Patients, in whom DVA in SIAR was observed, were more frequently female without reaching statistical significance (34.4 [26.9, 42.0] vs. 27.3 [24.7, 29.8]%, p = 0.066). The prevalence of DVA in SIAR was not significantly different in patients who presented with STEMI (see supplementary Table 4). In multivariable analysis, higher age (OR 1.44 per 10-year increase, 95% CI 1.15 to 1.80, p = 0.001) and right FAA (OR 2.52, 95% CI 1.72 to 3.69, p < 0.001) remained independent predictors of DVA in SIAR (see Fig. 3).

To further evaluate differences in the prevalence of DVA between both arms, patients who had left and right FAA either in the same or a subsequent procedure during the study period, were included for intraindividual comparison. In RUBR, DVA could be evaluated bilaterally in 30 patients during the same procedure and in 95 patients during a subsequent procedure. In 52 cases the same operator evaluated DVA in RUBR bilaterally and the average time span between evaluations was 31 ± 52 days, with a maximum of 273 days. DVA was significantly more prevalent in left RUBR (OR 2.7, 95% CI 1.1 to 7.6, p = 0.029) while no statistically significant differences could be found for 2nd degree DVA (OR 1.0, 95% CI 0.33 to 3.1, p = 1.0).

In SIAR, DVA could be evaluated bilaterally in 13 patients during the same procedure and in 94 patients during a subsequent procedure. In 34 cases the same operator evaluated DVA in SIAR bilaterally and the average time span between intraindividual evaluations was 36 ± 55 days, with a maximum of 273 days. DVA (OR 4.0, 95% CI 1.8 to 10.0, p < 0.001) and 2nd degree DVA (OR 5.5, 95% CI 1.2 to 51.1, p = 0.022) were significantly more prevalent in right SIAR respectively. Matched pairs contingency tables are provided in Tables 3 and 4.

**Table 3:**
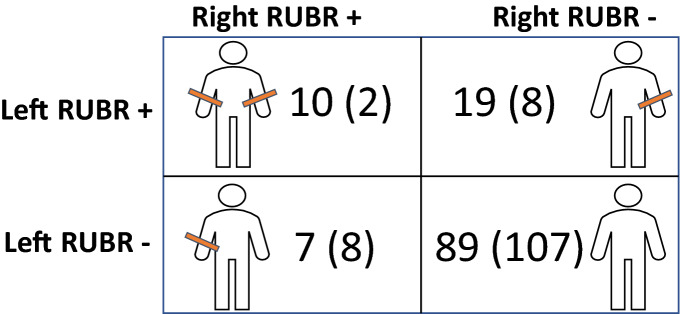
Intraindividual analysis of DVA in right vs. left RUBR.

In 125 patients, difficult vascular anatomy (DVA) could be assessed bilaterally in RUBR, either during the same procedure (n = 30) or during subsequent procedures (n = 95) allowing for intraindividual comparisons. Pairwise counts represent DVA and 2nd degree DVA (in brackets).

*RUBR* radial-ulnar-brachial region.

**Table 4:**
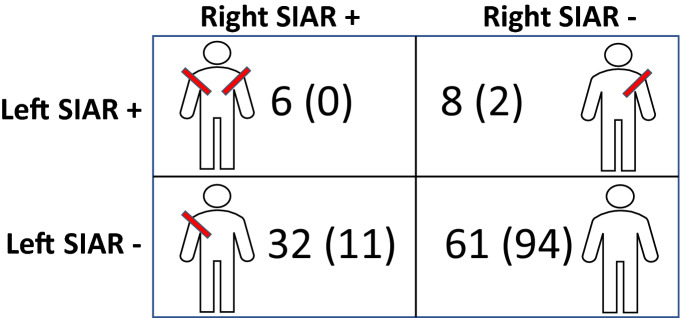
Intraindividual analysis of DVA in right vs. left SIAR.

In 107 patients, difficult vascular anatomy (DVA) could be assessed bilaterally in SIAR, either during the same procedure (n = 13) or during subsequent procedures (n = 94) allowing for intraindividual comparisons. Pairwise counts represent DVA and 2nd degree DVA (in brackets).

*SIAR* subclavian-innominate-aortic region.

## Discussion

Our retrospective single-center analysis of an all-comer cohort of patients, showed that the prevalence of DVA was relevant, ranging from 0.7 to 2.5% depending on the anatomical location. To our knowledge, this is the first study to quantify the prevalence of DVA especially in RUBR and separately for both arms.

2nd degree DVA was more prevalent in RUBR compared to SIAR despite not reaching statistical significance (2.0 vs. 1.5%). This is surprising, since most of previous studies exclusively focused on the assessment of DVA in SIAR. For instance, Rigatelli et al. observed a “hostile subclavian artery”, which could not be passed despite a sophisticated mother-and-child technique, in 2.1% of cases^6^. Studies exploring vascular variants in RUBR, predominantly focused on visual rather than functional assessment^5,13^ or almost exclusively focused on right RUBR^4,17^. The particular interest in vascular variants in SIAR might be attributed to the fact that abnormalities in SIAR, even in the less severe form of 1st degree DVA, can significantly hinder subsequent guidewire/guide catheter manipulation and is even associated with vascular injuries and thromboembolic stroke due to excessive device manipulation^6,9^. This is in stark contrast to 1st degree DVA in RUBR, which once this anatomical region is passed, does not subsequently interfere with procedural success in our experience. However, our data indicates that DVA in RUBR must be taken seriously. In this context, it is particularly noteworthy that 2nd degree DVA was never observed during TUA but only during TRA. This resonates with previous studies, which reported that the ulnar artery, as the natural continuation of the brachial artery, following a straight course at the forearm without excessive tortuosity, loops or unfavorable branching angles represents an at least equivalent alternative to TRA^18^. However, the proportion of transulnar access attempts in our study was insufficient to provide generalizable conclusions.

According to our knowledge, the prevalence of FAA failure due to bilateral 2nd degree DVA has not yet been systematically studied. Previous studies gave estimates of approximately 8% for bilateral anatomical variants focusing on angiographic descriptions of vascular variants rather than functional aspects^5^. With our quasi-all-comer-design and a high primary FAA rate of 97.6%, we are confident to sufficiently estimate the prevalence of bilateral 2nd degree DVA at 0.9%. The probability of encountering 2nd degree DVA at the contralateral arm, when 2nd degree DVA caused FAA failure at the ipsilateral arm, was found to be 15.4%. This indicates that DVA possibly clusters in individual patients, which was previously presumed by others^5,6^. However, this also implies that the presence of unilateral 2nd degree DVA should not discourage operators from subsequent FAA attempts at the contralateral arm before crossover to TFA.

The pathophysiology of DVA and its clinical predictors are not yet fully understood. In our study, independent predictors of DVA in RUBR were advanced age, lower height, and left FAA. In SIAR, independent predictors were limited to advanced age and right FAA. These findings concur well with previous studies, which identified advanced age and short stature as predictors for both severe tortuosity of the right subclavian artery^10^ and TRA failure^9,19^. In contrast to a previous study by Cha et al.^10^, which identified female gender and higher body mass index as predictors of severe tortuosity of the right subclavian artery, female gender and higher BMI were not found to be independent risk factors of DVA in our study population. This observation is of high importance for clinical practice, since both female sex and obesity are linked with increased risk of bleeding complications during transfemoral access^20,21^.

In multivariable analysis, the choice of access site (left vs. right FAA) stood out as the single most important predictor for DVA both in RUBR and SIAR. While earlier studies found that severe tortuosity of the subclavian artery and vascular variants in general were more prevalent at the right arm^5,6^, the access site was mostly not taken into account when DVA was analyzed in previous studies^10,17^.

Our results from intraindividual comparisons provide further evidence that DVA in general and 2nd degree DVA is more frequently found in right vs. left SIAR. These functional results match well with the aforementioned, angiographic observations that severe tortuosity of the subclavian artery is more prevalent at the right compared to the left arm^5,6^. The fact that both DVA and 2nd DVA were less prevalent in left SIAR might affect access site preferences for FAA in future procedures.

The results from intraindividual comparisons also further confirmed that DVA but not 2nd degree DVA was more frequently found in left vs. right RUBR. The higher prevalence of DVA in left vs. right RUBR was mainly driven by a higher prevalence of 1st degree DVA (see Table 2). This could be attributed to the fact that the left forearm is angled inwardly for operator comfort after left FAA is established. Inward angulation of the forearm with concomitant elbow flexion might lead to coiling of the radial artery at the brachioradial junction possibly impeding guidewire advancement. Therefore, it might be useful to bring the left arm into a neutral position when DVA is encountered, to facilitate guidewire passage. However, this was not the objective of our retrospective analysis and further experimental studies are needed to estimate a potential benefit. The prevalence of DVA was not significantly different in STEMI cases, neither in RUBR nor SIAR. This indicates that fixed anatomical variants, rather than interventional technique or the presence of vasospasm, are the cause of DVA.

### Limitations

The main limitations of our study encompass a single-center cohort, a low proportion of female patients in the study cohort and a nonrandomized study design. Additionally, it cannot be ruled out that information from previous procedures influenced the operator’s choice of primary access site, potentially leading to selection bias.

## Supplementary Information

Supplementary Information.

## Abbreviations

ACS: Acute coronary syndrome
CAD: Coronary artery disease
DVA: Difficult vascular anatomy
EGFR: Estimated glomerular filtration rate
FAA: Forearm artery access
NSTEMI: Non-ST-elevation myocardial infarction
PCI: Percutaneous coronary intervention
RUBR: Radial-ulnar-brachial region
SIAR: Subclavian-innominate-aortic region
STEMI: ST-elevation myocardial infarction
TFA: Transfemoral access
TRA: Transradial access
TUA: Transulnar access
