# Supplementary material for: Prevalence and predictors of difficult vascular anatomy in forearm artery access for coronary angiography and PCI
Source: Sci Rep. 2022 Jul 29;12:13060. doi: 10.1038/s41598-022-17435-1 (PMC9338070; doi:10.1038/s41598-022-17435-1)
Supplement: Supplementary file 1 — Supplementary Information. [file 41598_2022_17435_MOESM1_ESM.docx]

Prevalence and predictors of difficult vascular anatomy in forearm artery access for coronary angiography and PCI

# Roeschl Tobias^1^, MD, Jano Anas M.^1^, Fochler Franziska^1,2^ MD, Grewe Mona M^1,2^, Wacker Marlis^2^, Meier Kirstin^1^, MD, Schmidt Christian^1^, MD, Maier Lars^2^, MD, Grewe Peter H^1^, MD

^1^Clinic of Cardiology and Angiology, Klinikum Neumarkt, Neumarkt, Germany

^2^Department of Internal Medicine II, University Hospital Regensburg, Regensburg, Germany

No conflict of Interest for all authors

**Adress for Correspondence**

Peter H. Grewe, Clinic for Cardiology, Klinikum Neumarkt

Orcid i.d.: **0000-0001-9209-040X**

E-mail: [Peter.Grewe@Klinikum.Neumarkt.de](mailto:Peter.Grewe@Klinikum.Neumarkt.de)

# Supplementary

Supplementary figure 1: Flowchart of the exclusion process

| **Variable** |  |
| --- | --- |
| Age (years) | 71.3 [61.2, 79.4] |
| Weight (kg) | 83.0 [73.0, 94.0] |
| Height (cm) | 171.8 ± 9.3 |
| Body Mass Index (kg/m^2^) | 28.4 ± 5.1 |
| Body Surface Area (m^2^) | 2.0 ± 0.2 |
| Female sex | 384 (28.3%) |
| Arterial hypertension | 1178 (86.9%) |
| Diabetes mellitus | 381 (28.1%) |
| Active smoking | 341 (25.1%) |
| eGFR (ml/min/1.73 m^2^) | 74.7 ± 27.3 |
| Percutaneous coronary intervention | 539 (39.7%) |
| Left main stem intervention | 35 (2.6%) |
| Coronary bifurcation intervention | 124 (9.2%) |
| Bypass graft PCI | 8 (0.6%) |
| Intracoronary imaging and/or physiological assessment | 310 (22.9%) |
| **Clinical indication** |  |
| Suspected CAD or chronic coronary syndrome | 1121 (82.7%) |
| Acute coronary syndrome (ACS) |  |
| ST-Elevation myocardial infarction (STEMI) | 91 (6.7%) |
| Non-ST-Elevation-ACS | 139 (10.3%) |
| Cardiogenic shock | 4 (0.3%) |
| Cardiac arrest | 1 (0.1%) |

Supplementary table 1: Baseline patient characteristics (n=1356)

Values are mean ± SD for continuous, normally distributed data, median [IQR] for continuous, non-normally distributed data and n (%) for dichotomous data. CAD: Coronary artery disease, PCI: Percutaneous coronary intervention, eGFR: Estimated glomerular filtration rate.

| **Left forearm artery access** | 745 (54.9%) |
| --- | --- |
| Distal radial artery | 459 (61.6%) |
| Proximal radial artery | 273 (36.6%) |
| Ulnar artery | 12 (1.6%) |
| Anterior interosseous artery | 1 (0.1%) |
| **Right forearm artery access** | 611 (45.1%) |
| Distal radial artery | 4 (0.7%) |
| Proximal radial artery | 593 (97.1%) |
| Ulnar artery | 14 (2.3%) |

Supplementary table 2: Primary forearm access sites (n=1356)

|  | **DVA (-)** | **DVA (+)** | **p-value** |
| --- | --- | --- | --- |
| **Age (years)** | 69.1 [68.5, 70.0] | 74.6 [72.7, 77.0] | <0.001 |
| **Weight (kg)** | 84.8 [83.8, 86.0] | 77.4 [74.5, 80.0] | <0.001 |
| **Height (cm)** | 172.3 [171.8, 173.0] | 166.6 [165.1, 168.0] | <0.001 |
| **Body Mass Index (kg/m^2^)** | 28.5 [28.2, 29.0] | 27.9 [26.9, 29.0] | 0.21 |
| **eGFR (ml/min/1.73 m^2^)** | 75.5 [74.0, 77.0] | 67.2 [62.8, 72.0] | <0.001 |
| **Left FAA** | 52.8 [50.0, 55.6] % | 70.1 [62.4, 77.7] % | <0.001 |
| **STEMI** | 6.8 [5.4, 8.2] % | 5.1 [1.4, 8.9] % | 0.46 |
| **Female sex** | 25.8 [23.4, 28.3] % | 48.5 [40.1, 56.9] % | <0.001 |
| **Arterial hypertension** | 86.6 [84.6, 88.5] % | 88.2 [82.8, 93.7] % | 0.59 |
| **Diabetes mellitus** | 28.5 [26.0, 31.1] % | 25.0 [17.7, 32.3] % | 0.37 |
| **Current smoking** | 25.9 [23.4, 28.4] % | 16.9 [10.6, 23.2] % | 0.022 |

Supplementary table 3: Baseline patient characteristics in procedures where difficult vascular anatomy (DVA) in RUBR was observed (+) or not (-)

Values are means or percentages with 95% confidence intervals. RUBR: Radial-ulnar-brachial region, FAA: Forearm artery access, STEMI: ST-elevation myocardial infarction, eGFR: Estimated glomerular filtration rate, eGFR: Estimated glomerular filtration rate.

|  | **DVA (-)** | **DVA (+)** | **p-value** |
| --- | --- | --- | --- |
| **Age (years)** | 69.1 [68.4, 70.0] | 73.6 [71.7, 76.0] | <0.001 |
| **Weight (kg)** | 84.3 [83.3, 85.0] | 83.0 [80.1, 86.0] | 0.37 |
| **Height (cm)** | 172.1 [171.6, 173.0] | 169.5 [168.1, 171.0] | 0.001 |
| **Body Mass Index (kg/m^2^)** | 28.4 [28.1, 29.0] | 28.8 [27.9, 30.0] | 0.42 |
| **eGFR (ml/min/1.73 m^2^)** | 75.2 [73.7, 77.0] | 70.6 [66.1, 75.0] | 0.047 |
| **Left FAA** | 57.0 [54.1, 59.8] % | 37.1 [29.4, 44.8] % | <0.001 |
| **STEMI** | 6.6 [5.2, 8.0] % | 6.6 [2.7, 10.6] % | 0.98 |
| **Female sex** | 27.3 [24.7, 29.8] % | 34.4 [26.9, 42.0] % | 0.066 |
| **Arterial hypertension** | 86.4 [84.4, 88.4] % | 89.4 [84.5, 94.3] % | 0.31 |
| **Diabetes mellitus** | 28.7 [26.1, 31.4] % | 25.8 [18.8, 32.8] % | 0.45 |
| **Active smoking** | 25.8 [23.3, 28.3] % | 19.9 [13.5, 26.2] % | 0.11 |

Supplementary table 4: Baseline patient characteristics in procedures where difficult vascular anatomy (DVA) in SIAR was observed (+) or not (-)

Values are means or percentages with 95% confidence intervals. FAA: Forearm artery access, STEMI: ST-elevation myocardial infarction.
